# Expression of Follicle-Stimulating Hormone Receptor in Von Hippel-Lindau Associated Tumors and Cysts: An Immunohistochemical Study

**DOI:** 10.15586/jkc.v12i3.409

**Published:** 2025-09-24

**Authors:** Nicolae Ghinea, Maximilian Frosch, Anne Couvelard, Sefer Elezkurtaj, Vinciane Rebours, Philippe Camparo, Bertrand Guillonneau, Christine Julia Gizaw, Jan-Helge Klingler

**Affiliations:** 1 Institut National de la Santé et de la Recherche Médicale, Paris, France;; 2 FSHR-Theranostics, 11 Rue de Rungis, 75013 Paris, France;; 3 Institute of Neuropathology, University of Freiburg, Faculty of Medicine, Germany;; 4 Hôpital Bichat, Service de Pathologie, 46 rue Henri-Huchard, 75018 Paris;; 5 Institut für Pathologie Charité, Universitätmedizin Berlin; Campus Charité Mitte, Charitéplatz 1,10117 Berlin;; 6 Hôpital Beaujon, Service de Pancréatologie, 100 Boulevard du général Leclerc, 92110-Clichy, France;; 7 I-patH, 11 Allée de l’échauguette, 80000 Amiens, France;; 8 Sektionleiter uro-onkologische Chirurgie Klinik für Urologie Charité Universitätmedizin Berlin, Charitéplatz 1,10117 Berlin, Germany;; 9 Department of Neurosurgery, Medical Center, University of Freiburg, Faculty of Medicine, University of Freiburg, Germany France

**Keywords:** ccRCC, CNS-Hemangioblastoma, Kidney Cancer, panNET, VHL

## Abstract

Von Hippel-Lindau (VHL) disease is a hereditary condition caused by mutations in the *VHL*-tumor suppressor gene leading to constitutive overproduction of HIF-1alpha and HIF-2alpha, two proangiogenic factors, involved in the development of highly vascular tumors. Published evidence has shown that FSH-receptor is expressed in endothelial cells of blood vessels (BV) in several types of tumors. Given that VHL-associated tumors are highly vascular, it is plausible that FSH-receptor could be expressed in their vasculature as well. This immunohistochemical study involved 71 patients diagnosed with VHL-associated tumors, who required surgical intervention. Tissue specimens from these patients included CNS-hemangioblastoma, pancreatic neuroendocrine tumors (panNET), and clear cell renal cell carcinoma (ccRCC). Immunohistochemical staining was performed using a highly specific monoclonal antibody against the human FSH-receptor to assess its expression in the endothelial cells and tumor cells. The distribution of FSH-receptor staining was analyzed using digital imaging techniques. FSHR-protein expression was detected in the BV endothelial cells in 100% of VHL-associated CNS-hemangioblastoma, panNET, and ccRCC cases. In CNS-hemangioblastoma, 96% of cases showed FSH-receptor positivity in tumor stromal cells. In panNET, 88% of the cases displayed FSH-receptor expression in tumor cells. No tumor cells showed FSH-receptor expression in ccRCC. This is the first study to demonstrate FSH-receptor expression by cells of VHL-associated tumors, with distinct expression patterns in different tumor types.

## Introduction

Follicle-stimulating hormone (FSH), a central hormone of mammalian reproduction, is a glycoprotein produced in the anterior pituitary gland, and the classical target organs are the ovary and testis. FSH exerts its biological role by binding to FSH-receptor (FSHR). FSHR exists as four alternatively spliced isoforms of which three are cell surface membrane glycoproteins: FSHR1, FSHR2, and FSHR3, and a soluble binding protein, FSHR4 (1). Only FSHR1 and FSHR3 have known biological functions (2, 3).

FSHR1, the full length form of FSHR (mature glycoprotein protein of 678 amino acids, Mr=87 kDa), is a G-protein-coupled receptor. It possesses the distinctive pattern of seven transmembrane spanning domains and has a large extracellular domain responsible for the high-affinity binding of FSH. This extracellular domain is encoded by the first nine exons of *FSHR* gene. The last exon10 encodes both the transmembrane and the intracellular domains (4). FSHR1 through the PKA pathway mediates the FSH functions via the activation of adenylate

cyclase. In adult humans and animals, FSHR1 is mainly expressed in ovarian granulosa cells, testicular sertoli cells (SC), and minimally expressed on the endothelium lining the gonadal BV (5). In testis, the endothelial FSHR1 mediates translocation of FSH across the blood–testis barrier by a process of receptor-mediated transcytosis (6). In nonpregnant women, FSHR1 is also expressed by endothelial cells of BV supplying extragonadal reproductive tissues including endometrium, myometrium, and cervix (7). Published evidence supports ectopic and functional expression of FSHR1 in the angiogenic vasculature associated with several types of cancer (5, 8–14) and with various inflammatory diseases including benign prostatic hyperplasia (5), endometriosis (15, 16), and human atherosclerotic plaques (17).

FSHR3, a truncated isoform (exons 1–8), has an extracellular hormone-binding domain of 242 amino acids and behaves as a growth factor type 1 receptor that acts through calcium signaling and the MAPK/ERK pathway. It is predominant in ovarian surface epithelial cells and cancer cells. FSH/FSHR3-stem cell interaction is responsible for ovarian cancer cell proliferation (2).

Von Hippel-Lindau (VHL) disease is a hereditary condition caused by mutations in the *VHL* gene that lead to the development of vascular tumors such as clear cell renal cell carcinoma (ccRCC), central nervous system (CNS)-hemangioblastoma, and pancreatic neuroendocrine tumors (panNET). These tumors are characterized by excessive vascularization because of dysregulated hypoxia-inducible factor-1alpha signaling, which is central to VHL pathology (18). Because VHL-associated tumors are highly vascular and characterized by abnormal angiogenesis, it is plausible that FSHR1 is expressed in their vasculature as well.

The idea to check FSHR1 expression in VHL-associated tumors originated during our earlier work (5), where we first described the ectopic expression of FSHR1 on the endothelial cells of tumor BV. Among the various cancer types we analyzed in that study, we noticed immunohistochemical evidence of FSHR1 expression in a VHL-associated panNET lesion, though this observation was not explored at that time. The current study is a direct continuation of that original observation.

We conducted an immunohistochemical study to assess FSHR1-expression in VHL-associated tumors including ccRCC, CNS-hemangioblastoma, and panNET.

## Materials and Methods

### Tissue specimens

The present study included 71 patients diagnosed with VHL-associated tumors (41 (58%) were women and 30 (42%) were men (Table 1)). All the patients required surgical intervention. The specimens were fixed in formalin and embedded in paraffin. Paraffin sections (5 µm, 1.5–2.5 cm^2^) of tumors and cysts that occur as part of VHL disease were obtained from the biorepositories of the Institute of Neuropathology, Freiburg, Germany (42 patients with VHL-associated hemangioblastoma), “Charité” Hospital, Berlin, Germany (12 patients with VHL-associated ccRCC and cysts), and “Beaujon” Hospital, Clichy, France (17 patients with VHL-associated panNET). Paraffin sections for normal human testis tissue used as a positive control (three donors) were from the Biorepository of Lariboisière Hospital, Paris. Nontumoral tissue situated at 10 mm from the tumor borders that routinely accompanies surgical tumor samples present on the paraffin sections served as negative control. Four study investigators performed histologic analysis for each tumor specimen.

**Table 1:** Patients’ characteristics.

Tumor type	Location	Patients			
		Females		Males	
		Number	Age	Number	Age
CNS-Hemangioblastoma	Cerebellum	10	46(17-67)	14	48(21-58)
	Spinal cord	6	2(19-55)	6	9(19-50)
	Intradural/extramedular	3	56(25-73)	2	49(44-55)
	Brain stem	1	62	0	0
panNET	Pancreas	12	5(8-55)	5	43(27-59)
ccRCC	Kidney	9	47(37-63)	33	37(34-55)

* Median age

### Ethical approval

The study was performed in accordance with the Declaration of Helsinki and received ethical approval from the Institutional Review Board (IRB) 00006477 of HUPNVS, Paris 7 University, AP-HP, Paris, France, and Research Ethics Committee at the University Freiburg Medical Center (approval number 10008/09). All patients and donors have given written informed consent to the operation and the use of samples for research.

### Antibodies

The mouse anti-human FSHR antibody 323 was purified from a Hybridoma (American Type Culture Collection number CRL-2689) purchased in 2004. The mouse anti-hFSHR1 monoclonal antibody A02 (RRID: AB_3677298) was supplied by FSHR-Theranostics (Paris, France). FSHR1A02 recognizes an epitope present only in hFSHR1 and absent in hFSHR3, LHR, and TSHR.

Goat-anti-mouse IgG-horseradish peroxidase (Sigma-Aldrich Catalog number A3673, RRID: AB_258426) and goat-anti-mouse IgG-Alexa 555 (Invitrogen, ThermoFisher Scientific Catalog Number A-21442; RRID: AB_2535845) were used as secondary antibodies in immunohistochemistry and in immunofluorescence microscopy, respectively.

### Chemicals

Diaminobenzidine (DAB) was from Dako A/S, Glostrup, Denmark. Goat serum, 3-amino-9-ethylcarbazole (AEC), and hematoxylin Gill solution n°3 were purchased from Sigma-Aldrich, Saint-Quentin Fallavier, France. DAPI (4’, 6-diamidino-2-phenylidole) was from Invitrogen, ThermoFisher Scientific.

### Immunohistochemistry

The present immunohistochemistry study was performed at Institut de Pathologie des Hauts-de-France, Amiens, France. Formaldehyde-fixed, paraffin-embedded (FFPE) sections (5 µm) were mounted on positively charged SuperFrost Ultra Plus/SuperFrost microscope slides (Thermo Scientific). Immunohistochemistry was implemented on the automated immuno-histochemical stainer (Leica Bond RX, Leica Biosystems). FFPE slides were deparaffinized within the staining instrument and immunostained according to the manufacturer’s guidelines. Antigen retrieval was performed by incubating slides at high temperature with 10 mM citrate buffer, pH = 6. Paraffin sections of human testis, VHL-associated tumors and cysts were immunolabeled with the mouse anti-hFSHR1 monoclonal A02 antibody (concentration: 0.2 µg/mL). Goat anti-mouse IgG (Fc-specific) coupled to horseradish peroxidase (Sigma, 1:200 dilution) was used as secondary antibody. As chromogen, we used 3-amino-9-ethylcarbazole (AEC) and 3,3’-diamino- benzidine (DAB). The hFSHR1 expression by blood microvessels and tumor cells has been analyzed on digital images from whole images of sections obtained by using the high-throughput “Lamina slide scanner” from Akoya Perkin Elmer, available in Cochin Institute, Paris. The virtual slides were observed with Case Viewer, a specific software downloadable free of charge from the internet.

### Immunofluorescence microscopy

L-cells permanently expressing the hFSHR1 (19) were fixed in 4% paraformaldehyde, permeabilized in 0.1% Triton X-100 in PBS, stained for indirect fluorescence, and incubated with mouse anti-hFSHR1 monoclonal antibody A02 (30 ng/mL in goat serum-PBS). Goat anti-mouse IgG-Alexa 555 (dilution 1:750) has been used as secondary antibody. The cells were analyzed using an Olympus BX43 microscope with 20× and 40× objectives and an Olympus SC100 digital camera.

### EC50 determination of mouse anti-hFSHR1 monoclonal antibodies 323 and A02

In order to characterize and to check whether the mouse anti-hFSHR monoclonal antibody A02 retains specificity for hFSHR and whether it binds to this receptor with higher affinity than the parental FSHR323, a binding fluorescence-activated cell sorting (FACS) experiment using different antibody concentrations and hFSHR-L cells, WT-L-cells, and L-cells expressing hLHR was performed. The binding of the mouse anti-hFSHR monoclonal antibodies 323 and A02 to the cells was detected by using an anti-mouse IgG conjugated to APC (BD Bioscience).

### In situ hybridization

To localize the mRNA encoding for hFSHR1 within the endothelial cells of BV nourishing the VHL-associated ccRCC tumors (paraffin-embedded tissues), we used a 45-mer cDNA antisense oligonucleotide (5’-AAT-CCA-GCC-CAT-CAC-CAT-GAT-ACT-GGC-AGC-ATG-GCG-GAG-CTG-CAC-3’) complementary to the 1441–1486 base pair region of the FSHR1 sequence (5, 20). The 3’-biotinylated oligonucleotide probe (Eurogentec; Seraing, Belgium) was detected with alkaline phosphatase conjugated streptavidin (DakoCytomation In Situ Hybridization Detection System; Code K0601). The sites of hybridization were visualized by the colorimetric reaction of the enzyme conjugate with a 5-bromo-4-chloro-3-indolyl phosphate and nitro blue tetrazolium mixture. The tissues were counterstained by immersing slides in 0.5% methyl green in 0.1 M sodium acetate buffer, pH4.2.

## Results

### Expression of FSHR1 by cells in VHL-associated tumors: Antibodies’ validation

The FSHR1-isoform shares structural similarities with other two glycoprotein hormone receptors, namely, hLHR and hTSHR. Therefore, highly specific antibodies should avoid cross-reactivity with these receptors. By using viral-like particles of hFSHR1, hTSHR, and hLHR generated by expression of hFSHR1 (aa16-695), hTSHR (aa21-764), and hLHR (aa27-699) in HEK293 cells, Möker *et al*. (12) have demonstrated in ELISA studies that this is the case for the mouse anti-hFSHR monoclonal antibody 323. In their hands, this antibody was able to detect the receptor in immunohistochemical settings on paraffin-embedded Flp-In Chinese hamster ovary (CHO)/FSHR cells, and on paraffin-embedded human testicular SC.

**Figure 1:**
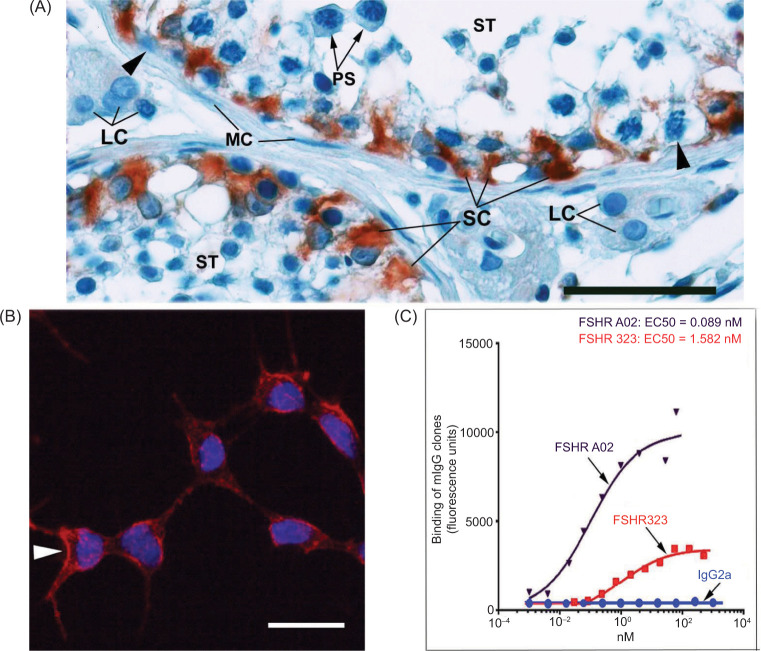
Immunohistochemical detection of FSHR1 in human testis and cultured L-cells permanently expressing the hFSHR1 receptor. (A) Immunohistochemical analysis was performed on paraffin-embedded sections of normal human testis tissue with the use of the anti-FSHR1 monoclonal antibody A02, followed by a secondary peroxidase-coupled antibody visualized with the use of the AEC red-brown peroxidase reaction product. Immunoreactivity was noticed in Sertoli cells (SC) of seminiferous tubules (ST). As expected, there was no signal for FSHR1 in spermatogonia (arrowheads), pachytene spermatocytes (PS), interstitial Leydig cells (LC), and in myoepithelial cells MC). Bar: 50 µm. (B) Immunofluorescence analysis performed on L-cells permanently expressing FSHR1, indicating cell surface expression of the receptor (arrowhead). The binding of the mouse anti-human FSHR1 monoclonal antibody A02 to FSHR1 was visualized with the use of the goat anti-mouse IgG-Alexa 555 (red fluorescence). Bar: 50 µm. (C) EC50 determination of FSHR323 and FSHR1A02 in hFSHR-L cells. The FSHR1A02 antibody retains specificity for hFSHR1 and binds to this receptor with higher affinity than the parental FSHR323. No binding was noted for an irrelevant mouse IgG2a antibody.

To examine the expression pattern of FSHR1 on paraffin-embedded VHL-associated tumors, we used a clone of the maturated monoclonal antibody FSHR323, the mouse anti-hFSHR1 monoclonal antibody A02. As illustrated in Figure 1, the FSHR1A02 antibody had the ability to stain hFSHR1 expressed by SC (the target cells of FSH) (panel A) and the cell surface hFSHR1 expressed in transfected L-cells (panel B**)**. FSHR1A02 antibody showed no binding to L-cells expressing hLHR or to WT L-cells used as negative controls. Screening FACS assays indicated that when tested for binding to hFSHR-L cells, the mouse anti-hFSHR1 monoclonal antibody A02 showed 18-fold improved EC50 value compared to the parental mouse anti-hFSHR1 monoclonal antibody 323 (panel C). These data, together with the design of FSHR1A02 antibody to target an epitope absent from hFSHR3, hLHR, and hTSHR, support its use as a specific detector of hFSHR1 in tissue sections.

### FSHR1 expression in VHL-associated ccRCC and renal cysts

**Figure 2:**
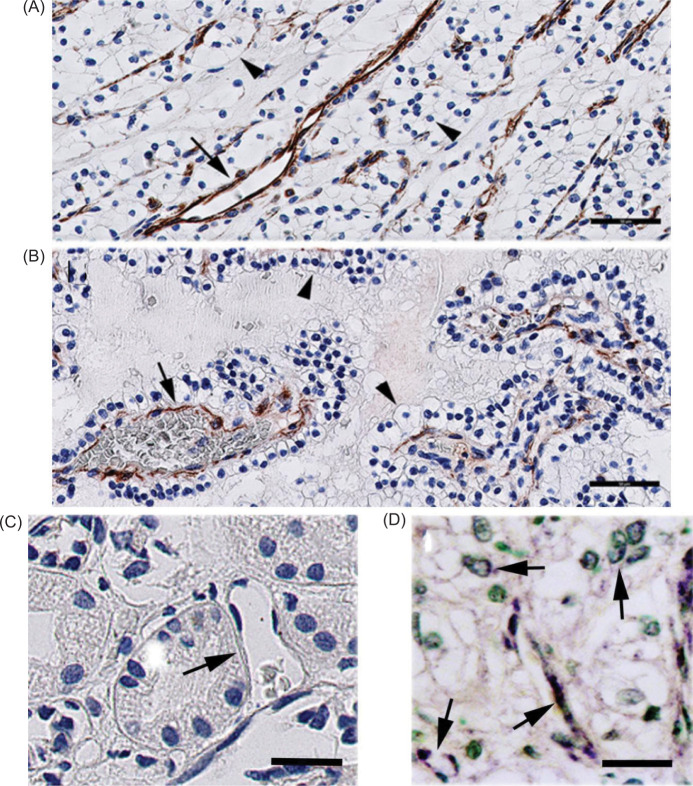
FSHR1 expressions in VHL-associated ccRCC tumors and renal cysts. (A,B) ccRCC and renal cysts, respectively. FSHR1 is detectable in blood vessel endothelial cells (arrows). By contrast, the epithelial cells are FSHR1-negative (arrowheads). (C) Normal kidney tissue with negative blood vessels for the FSHR1 (arrow). (D) In situ hybridization confirmation of the identity of the antigen recognized in tumor blood vessels (BV) by the FSHR1A02 antibody. FSHR1-mRNA was revealed with a biotinylated probe that was detected by a streptavidin–alkaline phosphatase conjugate, and visualized by the purple phosphatase-reaction product. The sections were counterstained with methyl green. Bars: 50 µm.

The endothelial cells of BV expressed FSHR1 in 100% of VHL-patients with ccRCC (Figure 2, panel A) and renal cysts (Figure 2, panel B**)**. No signal for FSHR1 was detected in normal kidney tissue (Figure 2, panel C). *In situ* hybridization confirmed the identity of the antigen recognized in tumor BV by the FSHR1A02 antibody (Figure 2, panel D). No FSHR1-positive tumor cells were visible in sections representing VHL-associated ccRCC (Figure 2, Table 2).

**Table 2:** FSHR1 expression in VHL-associated tumors.

Tumor type	Location	Patients with FSHRl expression in	
		Blood Vessels m positive cases/n all cases (%)	Tumor cells m positive cases/n all cases (%)
CNS-Hemangioblastoma	Cerebellum	24/24 (100)	23/24 (96)
	Spinal cord	12/12 (100)	12/12 (100)
	Brain stem	1/1 (100)	1/1 (100)
	lntradural	5/5 (100)	5/5 (100)
panNET	Pancreas	17/17 (100)	15/17 (88)
ccRCC	Kidney	12/12 (100)	0/12 (0)

### FSHR1 expression in VHL-associated CNS-hemangioblastoma

**Figure 3:**
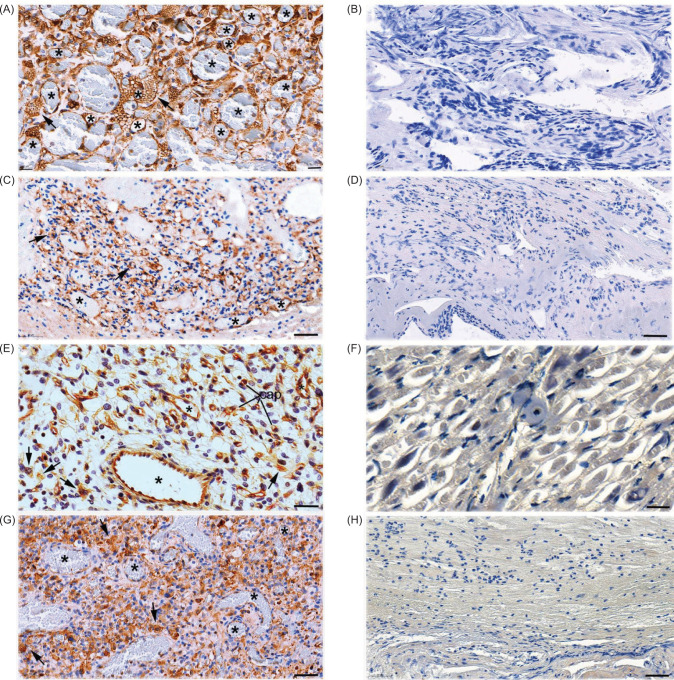
FSHR1 expression in VHL-associated CNS-hemangioblastoma. Immunohistochemical analysis was performed on paraffin-embedded sections of hemangioblastoma tissues with the use of the FSHR1A02 antibody, followed by a secondary peroxidase-coupled antibody visualized with the use of the brown peroxidase-reaction product of DAB. Sections were also stained with hematoxylin. Strong signal for FSHR1 was detectable in the endothelial cells of tumor’s large blood vessels (asterisks) and capillaries (arrowheads) supplying the VHL-associated hemangioblastoma located in cerebellum (A), brain stem (C), spinal cord (E), and intradural/extramedular (G). Tumor stromal cells (arrows) were also stained for FSHR1, especially in hemangioblastoma located in cerebellum, brain stem, and spinal cord. No FSHR1 was detectable in the normal tissues at the periphery of hemangioblastoma tumors located in cerebellum (B), brain stem (D), spinal cord (F), and intradural/extramedular (H). Bars: 50 µm.

CNS-hemangioblastoma is a benign highly vascular tumor. Immunohistochemical staining revealed that FSHR1 is uniformly expressed in BV through the tumor in 100% of the cases with CNS-hemangioblastoma (Table 2) located in the cerebellum (Figure 3, panel A), in brain stem (Figure 3, panel C), spinal cord (Figure 3, panel E), and intradural/extramedular (Figure 3, panel G). Tumor stromal cells with prominent vacuolization were also positive for FSHR1, especially in the cerebellar hemangioblastoma (Figure 3, panel A) and brain stem (Figure 3, panel C). FSHR1-positivity was not visible in non-tumoral tissue that accompanied surgical CNS-hemangioblastoma tumor samples (Figure 3, panels B, D, F, and H).

### FSHR1 expression in VHL-associated panNET

**Figure 4:**
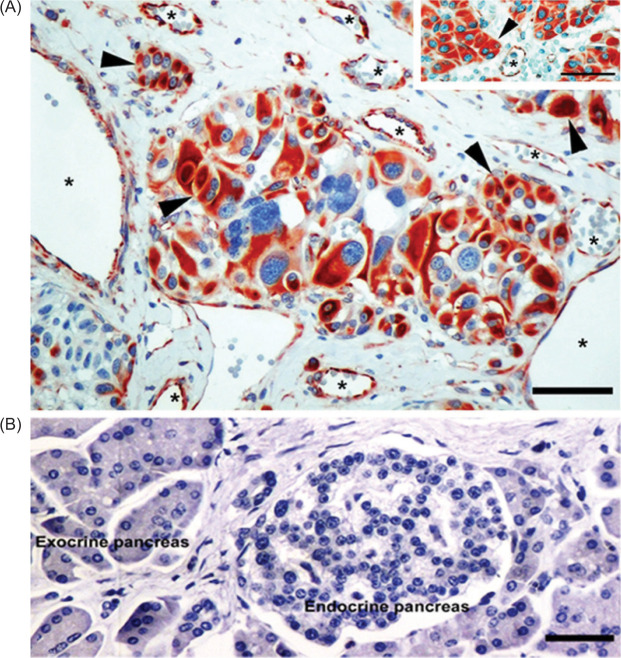
FSHR1 expression in VHL-associated pancreatic neuroendocrine tumors. The blood vessels (asterisks) and epithelial tumor cells (arrowheads) are FSHR1-positive (A). Similar staining was noticed when the FSHR1A02 antibody was replaced by the parent FSHR323 antibody (A, inset). No staining for FSHR1 was noted for normal human pancreas tissue (B). Bars: 50 µm.

FSHR1 also appears in BV endothelial cells lining the vasculature of 100% of VHL-patients with panNET. In these samples, FSHR1 was also detected in the tumor cells in 88% of the patients (Figure 4, panel A, and Table 2). No FSHR1 expression was visible in control normal pancreatic tissues that accompanied surgical tumor samples (Figure 4B).

## Discussion

FSHR has often been restricted to the reproductive system where its biological role was confined to gonads, ovarian granulosa cells, testicular SC, and BVBV supplying gonads. However, over the past decades, several studies from different laboratories reported the expression of FSH receptors in extra gonadal tissues mediating FSH activity there. Mariani *et al*. reported FSHR expression and cellular localization of FSHR in normal prostate, benign prostatic hyperplasia, and prostate cancer (21). Ben-Josef E *et al*. detected FSHR in hormone-refractary prostate cancer cells (22). By using different methods, including immunohistochemistry, immunofluorescence, electron microscopy, and immunoblotting on tissues from 1336 patients, Radu A *et al*. reported FSHR1 expression in endothelia of BV at the periphery of 11 types of tumors located in the prostate, ovary, testis, kidney, pancreas, liver, lung, stomach, colon, urinary bladder, and breast. Renal cell carcinoma was the only tumor type that showed FSHR1 in the BV throughout the tumor. Occasional cancer cells were also faintly stained only in prostate cancer, breast cancer, and in pancreatic cancer (5). Renner *et al*. detected FSHR1-positive BV in 11 types of soft tissue sarcomas (335 patients) and in tumor cells of all sarcoma subtypes examined (9). In these studies, endothelial FSHR1 expression is associated with tumor angiogenesis and vascular remodeling.

Several laboratories tried to trace the complex signaling pathways connecting FSH/FSHR to its ectopic expressions in various pathologies including cancer biology. RT-PCR analysis did not always find FSHR transcripts, and some scientists believe that the solution should be found by further investigating the expression of FSHR1 and FSHR3 isoforms and their specific role in pathological angiogenesis and cancer cell proliferation, respectively. At the same time, they point out the need for antibodies of high specificity (2).

Our recent findings in atherosclerosis reported FSHR1 expression in endothelial cells induced by oxidative stress generated by the accumulation of lipoproteins in the intima of large and medium arteries. In early stages of atherosclerosis, FSH/FSHR1 signaling, involved in the endothelial expression of VCAM1 upregulation, should have a positive role facilitating monocyte recruitment and diapedesis, a process beneficial for lipid clearance. In contrast, in later stages of the disease, FSHR1 expressed in macrophages has a negative role by driving VEGF secretion leading to angiogenesis and plaque instability (17). This dual role of FSHR1 signaling described in atherosclerosis could operate in tumors, as well. FSHR1 expression in tumor endothelial cells may initially support immune cell binding and infiltration into the tumor microenvironment potentially enhancing anti-tumor immunity. In advanced stages of cancer, FSHR1 expression in monocyte-derived tumor macrophages could promote pathological angiogenesis and immune suppression via VEGF.

Previous studies have shown that FSHR1 activation by FSH in ovarian granulosa cells (23) and in SC (24) leads to an increased level of hypoxia-inducible factor-1alpha, which results in upregulation of vascular endothelial growth factor (VEGF), a strong angiogenic factor. HIF-1alpha also upregulates the expression of VEGF in VHL-associated tumors (25). It is therefore reasonable to speculate that the same mechanism could induce VEGF/VEGFR2 signaling in tumor endothelial cells and thus promote angiogenesis in VHL-associated tumors.

Recent studies indicate that FSHR1 expression is influenced by TGF-beta1, a polypeptide factor involved in physiological (26) as well as in pathophysiological mechanisms of many diseases (27). In cancer, TGF-beta1 plays a dual role as a tumor suppressor in the early stages of the disease and contributes to angiogenesis and immune evasion in the advanced stages (28). To our knowledge, no studies have directly examined the relationship between endothelial FSHR1 expression and TGF-beta pathway activation in VHL-associated benign tumors, including CNS-hemangioblastoma. Future studies are needed to determine whether FSHR1 co-localizes with TGF-beta1 in tumor endothelial cells and whether such an association influences treatment resistance or immune modulation.

In this study, we show results from FSHR1 expression analysis in 71 samples of VHL-associated tumors representing ccRCC, panNET, and CNS-hemangioblastoma located in cerebellum, spinal cord, intradural/extramedullary, and brain stem. In each tumor analyzed, we detected FSHR1 expression by tumor endothelial cells throughout the tumor. A recent study confirms the presence of *FSHR* transcripts in multiple brain regions (29).

The VHL-associated tumors are typically benign, except for the VHL-associated ccRCC. Their treatment depends on several factors including type, size, location of the tumors, as well as the patient’s overall health and preferences. Common treatment options include surgical removal of the tumor and radiation therapy (30, 31). Recent publications suggest that a new drug, belzutifan, a hypoxia-inducible factor 2-alpha inhibitor shows promise as a therapeutic option for VHL-associated hemangioblastomas, particularly for patients not immediately requiring surgery (reviewed in 32–34).

Beyond its diagnostic relevance, FSHR1 expression may also hold predictive value for therapeutic response in VHL-associated tumors. In our previous work (35), we demonstrated that FSHR1 expression in the tumor-associated vasculature of primary ccRCC correlates strongly with clinical response to sunitinib, a VEGFR-targeted tyrosine kinase inhibitor, with high sensitivity and specificity, suggesting that FSHR1 may mark a functional angiogenic endothelium that is more susceptible to antiangiogenic therapy. This finding supports the potential use of FSHR1 not only as a biomarker for disease detection but also as a companion diagnostic tool to guide individualized therapy. Although our current study did not evaluate treatment response, the presence of FSHR1 in VHL-associated hemangioblastoma and other VHL-associated tumors could similarly reflect a vascular phenotype amenable to target inhibition.

Taken together, our results indicate that FSHR1 is not a ubiquitous or random marker but a highly restricted receptor normally confined to reproductive tissues. Its ectopic expression in tumor endothelium has been demonstrated in multiple studies (5–15). Its luminal expression on tumor BV makes FSHR1 accessible to circulating agents, and the anti-FSHR1 antibody A02 demonstrates selective binding and internalization. This supports FSHR’s role as a novel theranostic target, with potential for both imaging and target therapy in angiogenesis-driven tumors (36) such as those occurring in VHL disease. Future prospective studies are needed to validate this predictive capacity across the broader spectrum of VHL-associated tumors.

## Conclusion

This study provides the first evidence of FSHR1 expression in tumor BV and cells, in VHL-associated tumors.
